# Editorial: Smoldering Inflammation in Cardio-Immune-Metabolic Disorders

**DOI:** 10.3389/fphys.2021.651946

**Published:** 2021-03-19

**Authors:** Gilda Varricchi, Nazareno Paolocci, Felice Rivellese, Giuseppe Rengo

**Affiliations:** ^1^Department of Translational Medical Sciences, University of Naples Federico II, Naples, Italy; ^2^Center for Basic and Clinical Immunology Research (CISI), University of Naples Federico II, Naples, Italy; ^3^Institute of Experimental Endocrinology and Oncology (IEOS), National Research Council, Naples, Italy; ^4^Division of Cardiology, Department of Medicine, Johns Hopkins School of Medicine, Baltimore, MD, United States; ^5^Department of Biomedical Sciences, University of Padova, Padova, Italy; ^6^Centre for Experimental Medicine and Rheumatology, William Harvey Research Institute, Barts and the London School of Medicine and Dentistry, Queen Mary University of London, London, United Kingdom; ^7^Istituti Clinici Scientifici Maugeri SpA Società Benefit, Telese Terme, Italy

**Keywords:** allergic disorders, diabetes, obesity, low grade inflammation, sinovitis, IL-5, mast cells, Alzheimer's disease

“*If many remedies are prescribed for an illness, you may be certain that the illness has no cure.”*

Anton Chekhov - The Cherry Orchard -

Smoldering or low-grade inflammation plays a pivotal role in both physiological and pathological conditions (Calder et al., 2017; Zelechowska et al., 2018; Ronnback and Hansson, 2019). Aging is accompanied by a physiological decline in immune competence, termed immunosenescence, characterized by inflammaging (Antonelli et al., 2006; Franceschi et al., 2017; Varricchi et al., 2020b). Besides, low-grade inflammation is a prodrome of a variety of cardiometabolic disorders (Wen et al., 2012, van Greevenbroek et al., 2016), including obesity (Avalos et al., 2018; Trim et al., 2018), diabetes (Zatterale et al.), and cardiovascular diseases (Hoogeveen et al., 2018; Xu et al., 2019). Immune cells, strategically localized also in white adipose tissue (Horckmans et al., 2018; Zelechowska et al., 2018; Merrick et al., 2019; Plotkin et al., 2019), are an important source of pro-inflammatory cytokines in pathophysiological conditions (Varricchi et al., 2019a,b, 2020c; Marone et al., 2020). There is increasing awareness that specific biomarkers of smoldering inflammation are predictive of cardiovascular risks (Weber et al., 2004; Wolber et al., 2007; Varricchi et al., 2020a). Chronic low-grade inflammation also participates in the initiation and progression of several disorders of the immune system such as rheumatoid arthritis (Rivellese et al.; Siouti and Andreakos, 2019), psoriatic arthritis (Gisondi et al.; Girolomoni et al., 2017), and allergic diseases (Weiss, 2005; Pelaia et al., 2015; Canonica et al., 2016; Ferrando et al., 2017).

This Research Topic's driving force was to collect new acquisitions on the role of smoldering inflammation in diverse clinical pathological settings, examining them through the lens of temporal and spatial changes in immune cells and their products, such as cytokines.

Pucino et al. highlighted the interplay between metabolism, immunity and inflammation in patients with rheumatoid arthritis. The authors provided evidence that metabolic alterations of tissue microenvironment plays a pivotal role in the pathophysiology of rheumatoid arthritis. On the same ground, Moschetta et al. illustrated the role of inflammatory sinovitis in the development of hemophilic arthropathy. They discussed the role of imbalance of pro- and anti-inflammatory cytokines in inducing hemophilic arthropathy. The authors suggested that modulation of synovial inflammation could represent a novel therapeutic approach to prevent hemophilic arthropathy. Calcaterra et al. discussed the “two hits” hypothesis of synovitis in hemophilic arthropathy.

Rivellese et al. evaluated the possible contribution of synovial mast cells and their mediators to histological features of synovitis in severe and/or early rheumatoid arthritis. The authors demonstrated that disease-modifying anti-rheumatic drugs (DMARDs) reduced synovial inflammation and mast cell infiltration only in half of the patients examined. The presence of mast cells after 6 months of treatment with DMARDs was associated with a higher disease activity. They concluded that synovial mast cell are associated with disease severity. Gisondi et al. discussed the pathophysiological relationship between psoriasis, a chronic, systemic immune-mediated disease and cardiometabolic comorbidities and the therapeutic strategies to modulate low-grade inflammation in these patients.

It is well-established that several cytokines (e.g., IL-4, IL-5, and IL-13) (Varricchi and Canonica, 2016; Peters and Wenzel, 2020; Marone et al.) and alarmins (e.g., TSLP, IL-33, IL-25; Afferni et al., 2018; Varricchi et al., 2018; Marone et al., 2019; Porsbjerg et al., 2020) play a pivotal role in different phenotypes of asthma. Pelaia et al. extensively reviewed the central role of IL- 5 in the pathogenesis of severe eosinophilic asthma. The latter condition can be responsive to inhaled and/or systemic glucocorticoids that reduce eosinophilia (Hong et al., 2020).

However, severe eosinophilic asthma may be resistant to glucocorticoids and require therapies with specific monoclonal antibodies (mAbs), targeting IL-5/IL-5Rα (Varricchi and Canonica, 2016). Experimental models and clinical studies have demonstrated that IL-13 is an important cytokine in chronic airway inflammation. IL-13 is produced by human basophils (Gibbs et al., 1996; Ochensberger et al., 1996; Redrup et al., 1998; Patella et al., 2000; Genovese et al., 2003; Galeotti et al., 2019) and mast cells (Fushimi et al., 1998; Lorentz et al., 2000), the primary effector cells of allergic disorders (Marone et al., 2014; Varricchi et al., 2019c; Miyake et al., 2020). Marone et al. analyzed the biochemical and immunological effects of IL-13 in the context of experimental models of asthma and in asthmatic subjects. Despite promising results in several *in vitro* and *in vivo* models of allergic inflammation, the efficacy of mAbs anti-IL-13 in patients with asthma has been surprisingly negative (Hanania et al., 2016; Russell et al., 2018).

Obesity is one of the major health burdens of the twenty-first century as it contributes to insulin resistance and type 2 diabetes (Calay and Hotamisligil, 2013). Chronic, low-grade inflammation in adipose tissue is a crucial risk factor for the development of obesity and type 2 diabetes. Obesity is characterized by activation of the innate and adaptive immune system which may explain the increase susceptibility to develop metabolic disorders such as diabetes mellitus (Saltiel and Olefsky, 2017). Zatterale et al. carefully examined the molecular pathways linking obesity-induced inflammation and insulin resistance. The authors elegantly discussed the complex role of innate and adaptive immunity in obesity. Finally, they provided evidence that low-grade inflammation might represent a novel therapeutic target for metabolic diseases. Osteopontin produced by several immune cells, endothelial cells, and fibroblasts, is involved in cardiovascular diseases (Abdelaziz Mohamed et al., 2019; Vianello et al., 2020). Moschetta et al. reported that osteopontin is linked to pathological dysregulation of the arginine pathway in patients with coronary artery disease.

Alzheimer's disease (AD) is the most prevalent form of dementia in the elderly. A vast amount of literature indicates a role of inflammation in AD pathophysiology and several findings support the existence of a link between periodontitis, a chronic inflammatory oral disease and Alzheimer's disease (Heppner et al., 2015). Liccardo et al. provided an upgrade on the emerging evidence supporting a relationship between periodontitis and Alzheimer's disease.

But how do we protect ourselves from chronic inflammation? The above contributions highlight the need to counter clinical conditions that can determine the progression from acute to chronic inflammation. Among them, we should aim to lower cholesterol, reduce obesity, prevent gum disease, and stop smoking. Dietary changes are also likely to be important, including eliminating food and beverages high in fructose and other refined sugars while increasing our intake of polyphenols (Serino and Salazar, 2018), such as those contained in vegetables, fruits, and seeds. These alterations may represent an 'anti-inflammatory' lifestyle which may help reduce smoldering inflammation in chronic inflammatory conditions. Is this doable? In other words, does the multisource smoldering inflammation require many remedies? If so, then there might be no cure for this condition, and we have to side with Chekov on this one. In this scenario appears important to mention that results from the CANTOS trial have demonstrated that treatment with Canakinumab, a monoclonal antibody anti-IL-1β of patients with previous myocardial infarction and a high-sensitivity C-reactive protein level results in significantly reduced cardiovascular events. Moreover, patients with genetically-determined decreased IL-6 signaling showed a reduced risk of cardiovascular events and increased life-span (Rosa et al., 2019).

Early interventions, however, would help (e.g., a timely detection of any inflammatory focus). Pursuing this is feasible for easy-to-access areas of our body, such as skin, joints, and mouth. Moreover, preventing or eradicating the accumulation of visceral fat that is a consolidated fomite of chronic inflammation and atherosclerosis (Alexopoulos et al., 2014). Conversely, doing so for visceral organs is more complicated and requires a more articulated level of repeated inspections.

If chronic inflammation is, indeed, an enduring burning flame, then making an analogy to the fire of a match suggests another ineludible point (Figure 1). A match is composed of fuel (more specifically, antimony trisulfide) and an oxidant (an oxygen provider, i.e., potassium chlorate). We have described “antimony trisulfide” of different kinds (triggers and fuels), but we should not forget the importance of countering oxidative stress while cutting off the fuel and the trigger. Indeed, along with cytokines, reactive oxygen species can act as propagators of smoldering inflammation (Liu et al., 2020; Wiegman et al., 2020), morphing the phenomenon from local to systemic. We feel that this is another fertile and yet poorly explored terrain for future investigation.

**Figure 1:**
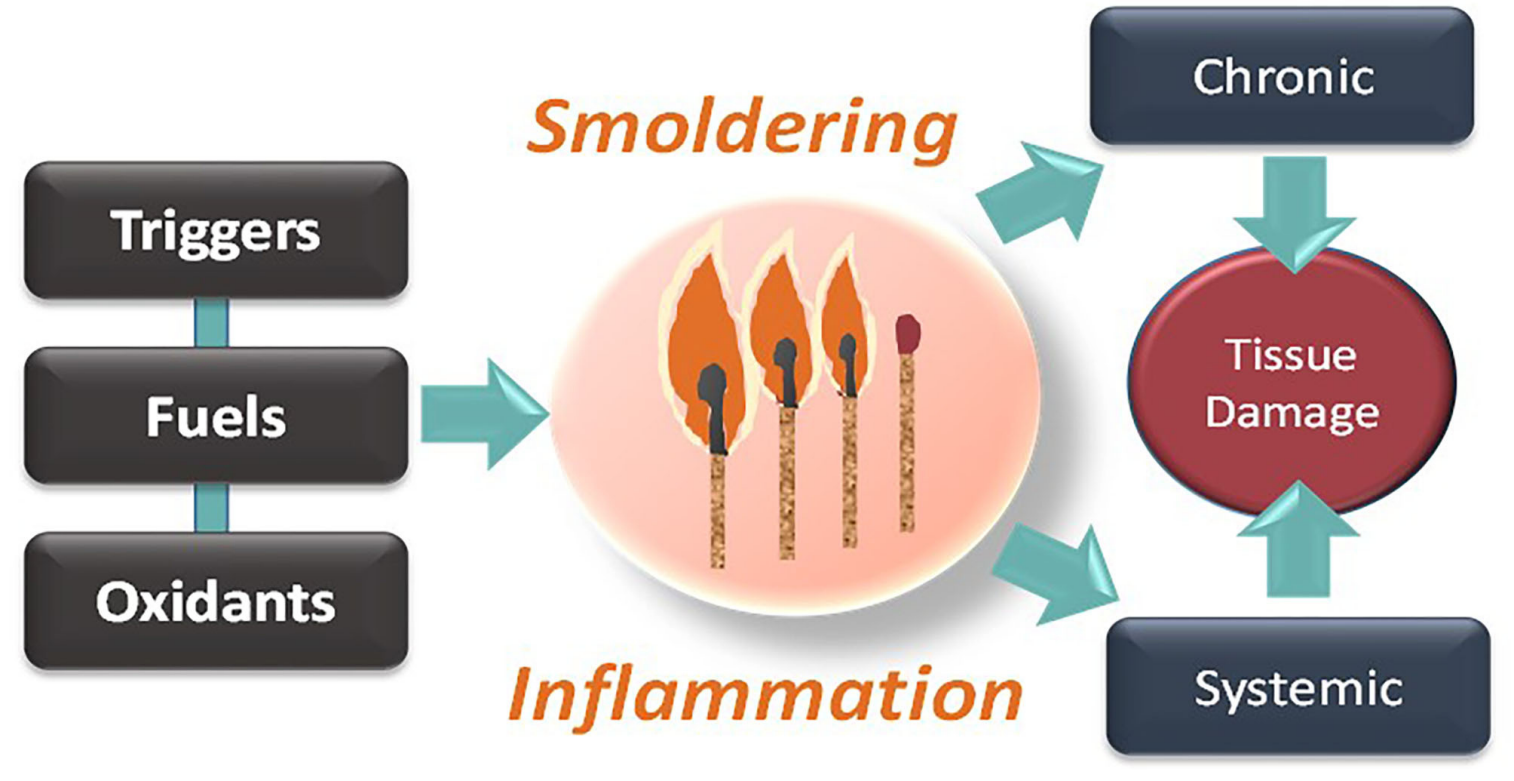
Smoldering inflammation as a progressive disease requiring triggers, fuels, and pro-oxidative conditions, ultimately resulting in systemic tissue and chronic damage.

We hope that articles harnessed in the current Research Topic help the readers with some new clues on how low-grade inflammation is initiated, maintained, and eventually resolved, at least to some extent.

## Author Contributions

GV wrote the article. NP, FR, and GR edited the article. GV, NP, and GR revised the article. All authors contributed to the article and approved the submitted version.

## Conflict of Interest

The authors declare that the research was conducted in the absence of any commercial or financial relationships that could be construed as a potential conflict of interest.

## References

[B1] Abdelaziz Mohamed I. Gadeau A. P. Hasan A. Abdulrahman N. Mraiche F. (2019). Osteopontin: a promising therapeutic target in cardiac fibrosis. Cells 8:1558. 10.3390/cells8121558 31816901 PMC6952988

[B2] Afferni C. Buccione C. Andreone S. Galdiero M. R. Varricchi G. Marone G.. (2018). The pleiotropic immunomodulatory functions of IL-33 and its implications in tumor immunity. Front Immunol. 9:2601. 10.3389/fimmu.2018.02601 30483263 PMC6242976

[B3] Alexopoulos N. Katritsis D. Raggi P. (2014). Visceral adipose tissue as a source of inflammation and promoter of atherosclerosis. Atherosclerosis 233, 104–112. 10.1016/j.atherosclerosis.2013.12.023 24529130

[B4] Antonelli A. Rotondi M. Fallahi P. Ferrari S. M. Paolicchi A. Romagnani P.. (2006). Increase of CXC chemokine CXCL10 and CC chemokine CCL2 serum levels in normal ageing. Cytokine 34, 32–38. 10.1016/j.cyto.2006.03.012 16697212

[B5] Avalos Y. Kerr B. Maliqueo M. Dorfman M. (2018). Cell and molecular mechanisms behind diet-induced hypothalamic inflammation and obesity. J Neuroendocrinol. 30:e12598. 10.1111/jne.12598 29645315

[B6] Calay E. S. Hotamisligil G. S. (2013). Turning off the inflammatory, but not the metabolic, flames. Nat Med. 19, 265–267. 10.1038/nm.3114 23467233

[B7] Calder P. C. Bosco N. Bourdet-Sicard R. Capuron L. Delzenne N. Dore J.. (2017). Health relevance of the modification of low grade inflammation in ageing (inflammageing) and the role of nutrition. Ageing Res. Rev. 40, 95–119. 10.1016/j.arr.2017.09.001 28899766

[B8] Canonica G. W. Senna G. Mitchell P. D. O'Byrne P. M. Passalacqua G. Varricchi G. (2016). Therapeutic interventions in severe asthma. World Allergy Organ. J. 9:40. 10.1186/s40413-016-0130-3 27942351 PMC5125042

[B9] Ferrando M. Bagnasco D. Varricchi G. Bernardi S. Bragantini A. Passalacqua G.. (2017). Personalized medicine in allergy. Allergy Asthma Immunol. Res. 9, 15–24. 10.4168/aair.2017.9.1.15 27826958 PMC5102831

[B10] Franceschi C. Garagnani P. Vitale G. Capri M. Salvioli S. (2017). Inflammaging and 'Garb- aging'. Trends Endocrinol. Metab. 28, 199–212. 10.1016/j.tem.2016.09.005 27789101

[B11] Fushimi T. Okayama H. Shimura S. Saitoh H. Shirato K. (1998). Dexamethasone suppresses gene expression and production of IL-13 by human mast cell line and lung mast cells. J. Allergy Clin. Immunol. 102, 134–142. 10.1016/S0091-6749(98)70064-8 9679857

[B12] Galeotti C. Stephen-Victor E. Karnam A. Das M. Gilardin L. Maddur M. S.. (2019). Intravenous immunoglobulin induces IL-4 in human basophils by signaling through surface-bound IgE. J. Allergy Clin. Immunol. 144, 524.e8–535.e8. 10.1016/j.jaci.2018.10.064 30529242

[B13] Genovese A. Borgia G. Bjorck L. Petraroli A. de Paulis A. Piazza M.. (2003). Immunoglobulin superantigen protein L induces IL-4 and IL-13 secretion from human Fc epsilon RI+ cells through interaction with the kappa light chains of IgE. J Immunol. 170, 1854–1861. 10.4049/jimmunol.170.4.1854 12574351

[B14] Gibbs B. F. Haas H. Falcone F. H. Albrecht C. Vollrath I. B. Noll T.. (1996). Purified human peripheral blood basophils release interleukin-13 and preformed interleukin-4 following immunological activation. Eur. J. Immunol. 26, 2493–2498. 10.1002/eji.1830261033 8898965

[B15] Girolomoni G. Strohal R. Puig L. Bachelez H. Barker J. Boehncke W. H.. (2017). The role of IL-23 and the IL-23/TH 17 immune axis in the pathogenesis and treatment of psoriasis. J. Eur. Acad. Dermatol. Venereol. 31, 1616–1626. 10.1111/jdv.14433 28653490 PMC5697699

[B16] Hanania N. A. Korenblat P. Chapman K. R. Bateman E. D. Kopecky P. Paggiaro P.. (2016). Efficacy and safety of lebrikizumab in patients with uncontrolled asthma (LAVOLTA I and LAVOLTA II): replicate, phase 3, randomised, double-blind, placebo-controlled trials. Lancet Respir Med. 4, 781–796. 10.1016/S2213-2600(16)30265-X 27616196

[B17] Heppner F. L. Ransohoff R. M. Becher B. (2015). Immune attack: the role of inflammation in Alzheimer disease. Nat. Rev. Neurosci. 16:358–372. 10.1038/nrn3880 25991443

[B18] Hong S. G. Sato N. Legrand F. Gadkari M. Makiya M. Stokes K.. (2020). Glucocorticoid- induced eosinopenia results from CXCR4-dependent bone marrow migration. Blood 136, 2667–2678. 10.1182/blood.2020005161 32659786 PMC7735160

[B19] Hoogeveen R. M. Nahrendorf M. Riksen N. P. Netea M. G. de Winther M. P. J. Lutgens E.. (2018). Monocyte and haematopoietic progenitor reprogramming as common mechanism underlying chronic inflammatory and cardiovascular diseases. Eur. Heart J. 39, 3521–3527. 10.1093/eurheartj/ehx581 29069365 PMC6174026

[B20] Horckmans M. Bianchini M. Santovito D. Megens R. T. A. Springael J. Y. Negri I.. (2018). Pericardial adipose tissue regulates granulopoiesis, fibrosis, and cardiac function after myocardial infarction. Circulation 137, 948–960. 10.1161/CIRCULATIONAHA.117.028833 29167227

[B21] Liu J. Li Y. Chen S. Lin Y. Lai H. Chen B.. (2020). Biomedical application of reactive oxygen species-responsive nanocarriers in cancer, inflammation, and neurodegenerative diseases. Front. Chem. 8:838. 10.3389/fchem.2020.00838 33062637 PMC7530259

[B22] Lorentz A. Schwengberg S. Sellge G. Manns M. P. Bischoff S. C. (2000). Human intestinal mast cells are capable of producing different cytokine profiles: role of IgE receptor cross-linking and IL-4. J. Immunol. 164, 43–48. 10.4049/jimmunol.164.1.43 10604991

[B23] Marone G. Borriello F. Varricchi G. Genovese A. Granata F. (2014). Basophils: historical reflections and perspectives. Chem. Immunol. Allergy 100, 172–192. 10.1159/000358734 24925398

[B24] Marone G. Rossi F. W. Pecoraro A. Pucino V. Criscuolo G. Paulis A.. (2020). HIV gp120 induces the release of proinflammatory, angiogenic, and lymphangiogenic factors from human lung mast cells. Vaccines 8:208. 10.3390/vaccines8020208 32375243 PMC7349869

[B25] Marone G. Spadaro G. Braile M. Poto R. Criscuolo G. Pahima H.. (2019). Tezepelumab: a novel biological therapy for the treatment of severe uncontrolled asthma. Expert. Opin. Investig. Drugs. 28, 931–940. 10.1080/13543784.2019.1672657 31549891

[B26] Merrick D. Sakers A. Irgebay Z. Okada C. Calvert C. Morley M. P.. (2019). Identification of a mesenchymal progenitor cell hierarchy in adipose tissue. Science 364:eaav2501. 10.1126/science.aav2501 31023895 PMC6816238

[B27] Miyake K. Shibata S. Yoshikawa S. Karasuyama H. (2020). Basophils and their effector molecules in allergic disorders. Allergy 10.1111/all.14662. [Epub ahead of print]. 33205439

[B28] Ochensberger B. Daepp G. C. Rihs S. Dahinden C. A. (1996). Human blood basophils produce interleukin-13 in response to IgE-receptor-dependent and -independent activation. Blood 88, 3028–3037. 10.1182/blood.V88.8.3028.bloodjournal8883028 8874201

[B29] Patella V. Florio G. Petraroli A. Marone G. (2000). HIV-1 gp120 induces IL-4 and IL-13 release from human Fc epsilon RI+ cells through interaction with the VH3 region of IgE. J. Immunol. 164, 589–595. 10.4049/jimmunol.164.2.589 10623799

[B30] Pelaia G. Vatrella A. Busceti M. T. Gallelli L. Calabrese C. Terracciano R.. (2015). Cellular mechanisms underlying eosinophilic and neutrophilic airway inflammation in asthma. Mediators Inflamm. 2015:879783. 10.1155/2015/879783 25878402 PMC4386709

[B31] Peters M. C. Wenzel S. E. (2020). Intersection of biology and therapeutics: type 2 targeted therapeutics for adult asthma. Lancet 395, 371–383. 10.1016/S0140-6736(19)33005-3 32007172 PMC8522504

[B32] Plotkin J. D. Elias M. G. Fereydouni M. Daniels-Wells T. R. Dellinger A. L. Penichet M. L.. (2019). Human mast cells from adipose tissue target and induce apoptosis of breast cancer cells. Front. Immunol. 10:138. 10.3389/fimmu.2019.00138 30833944 PMC6387946

[B33] Porsbjerg C. M. Sverrild A. Lloyd C. M. Menzies-Gow A. N. Bel E. H. (2020). Anti-alarmins in asthma: targeting the airway epithelium with next-generation biologics. Eur. Respir J. 56:2000260. 10.1183/13993003.00260-2020 32586879 PMC7676874

[B34] Redrup A. C. Howard B. P. MacGlashan D. W. Jr. Kagey-Sobotka A. Lichtenstein L. M. Schroeder J. T. (1998). Differential regulation of IL-4 and IL-13 secretion by human basophils: their relationship to histamine release in mixed leukocyte cultures. J. Immunol. 160, 1957–1964. 9469459

[B35] Ronnback C. Hansson E. (2019). The importance and control of low-grade inflammation due to damage of cellular barrier systems that may lead to systemic inflammation. Front. Neurol. 10:533. 10.3389/fneur.2019.00533 31191433 PMC6549124

[B36] Rosa M. Chignon A. Li Z. Boulanger M. C. Arsenault B. J. Bossé Y. (2019). Mendelian randomization study of IL6 signaling in cardiovascular diseases, immune-related disorders and longevity. NPJ Genom. Med. 4:23. 10.1038/s41525-019-0097-4 31552141 PMC6754413

[B37] Russell R. J. Chachi L. FitzGerald J. M. Backer V. Olivenstein R. Titlestad I. L.. (2018). Effect of tralokinumab, an interleukin-13 neutralising monoclonal antibody, on eosinophilic airway inflammation in uncontrolled moderate-to-severe asthma (MESOS): a multicentre, double-blind, randomised, placebo-controlled phase 2 trial. Lancet Respir Med. 6, 499–510. 10.1016/S2213-2600(18)30201-7 29793857

[B38] Saltiel A. R. Olefsky J. M. (2017). Inflammatory mechanisms linking obesity and metabolic disease. J. Clin. Invest. 127, 1–4. 10.1172/JCI92035 28045402 PMC5199709

[B39] Serino A. Salazar G. (2018). Protective role of polyphenols against vascular inflammation, aging and cardiovascular disease. Nutrients 11:53. 10.3390/nu11010053 30597847 PMC6357531

[B40] Siouti E. Andreakos E. (2019). The many facets of macrophages in rheumatoid arthritis. Biochem. Pharmacol. 165, 152–169. 10.1016/j.bcp.2019.03.029 30910693

[B41] Trim W. Turner J. E. Thompson D. (2018). Parallels in immunometabolic adipose tissue dysfunction with ageing and obesity. Front. Immunol. 9:169. 10.3389/fimmu.2018.00169 29479350 PMC5811473

[B42] van Greevenbroek M. M. Schalkwijk C. G. Stehouwer C. D. (2016). Dysfunctional adipose tissue and low-grade inflammation in the management of the metabolic syndrome: current practices and future advances. F1000Res. 5:F1000. 10.12688/f1000research.8971.1 27803798 PMC5070595

[B43] Varricchi G. Bencivenga L. Poto R. Pecoraro A. Shamji M. H. Rengo G. (2020b). The emerging role of T follicular helper (TFH) cells in aging: influence on the immune frailty. Ageing Res. Rev. 61:101071. 10.1016/j.arr.2020.101071 32344191

[B44] Varricchi G. Canonica G. W. (2016). The role of interleukin 5 in asthma. Expert Rev. Clin. Immunol. 12, 903–905. 10.1080/1744666X.2016.1208564 27450970

[B45] Varricchi G. de Paulis A. Marone G. Galli S. J. (2019c). Future needs in mast cell biology. Int. J. Mol. Sci. 20:4397. 10.3390/ijms20184397 31500217 PMC6769913

[B46] Varricchi G. Loffredo S. Bencivenga L. Ferrara A. L. Gambino G. Ferrara N.. (2020a). Angiopoietins, vascular endothelial growth factors and secretory phospholipase A2 in ischemic and non-ischemic heart failure. J. Clin. Med. 9:1928. 10.3390/jcm9061928 32575548 PMC7356305

[B47] Varricchi G. Loffredo S. Borriello F. Pecoraro A. Rivellese F. Genovese A.. (2019a). Superantigenic activation of human cardiac mast cells. Int. J. Mol. Sci. 20:1828. 10.3390/ijms20081828 31013832 PMC6514993

[B48] Varricchi G. Marone G. Kovanen P. T. (2020c). Cardiac mast cells: underappreciated immune cells in cardiovascular homeostasis and disease. Trends Immunol. 41, 734–746. 10.1016/j.it.2020.06.006 32605802

[B49] Varricchi G. Pecoraro A. Marone G. Criscuolo G. Spadaro G. Genovese A. (2018). Thymic stromal lymphopoietin isoforms, inflammatory disorders, and cancer. Front. Immunol. 9:1595. 10.3389/fimmu.2018.01595 30057581 PMC6053489

[B50] Varricchi G. Rossi F. W. Galdiero M. R. Granata F. Criscuolo G. Spadaro G.. (2019b). Physiological roles of mast cells: collegium internationale allergologicum update 2019. Int. Arch. Allergy Immunol. 179, 247–261. 10.1159/000500088 31137021

[B51] Vianello E. Kalousova M. Dozio E. Tacchini L. Zima T. Corsi Romanelli M. M. (2020). Osteopontin: the molecular bridge between fat and cardiac-renal disorders. Int. J. Mol. Sci. 21:5568. 10.3390/ijms21155568 32759639 PMC7432729

[B52] Weber M. Dill T. Arnold R. Rau M. Ekinci O. Muller K. D.. (2004). N-terminal B-type natriuretic peptide predicts extent of coronary artery disease and ischemia in patients with stable angina pectoris. Am. Heart J. 148, 612–620. 10.1016/j.ahj.2004.04.021 15459591

[B53] Weiss S. T. (2005). Obesity: insight into the origins of asthma. Nat. Immunol. 6, 537–539. 10.1038/ni0605-537 15908930

[B54] Wen H. Ting J. P. O'Neill L. A. (2012). A role for the NLRP3 inflammasome in metabolic diseases–did Warburg miss inflammation? Nat. Immunol. 13, 352–357. 10.1038/ni.2228 22430788 PMC4090390

[B55] Wiegman C. H. Li F. Ryffel B. Togbe D. Chung K. F. (2020). Oxidative stress in ozone-induced chronic lung inflammation and emphysema: a facet of chronic obstructive pulmonary disease. Front. Immunol. 11:1957. 10.3389/fimmu.2020.01957 32983127 PMC7492639

[B56] Wolber T. Maeder M. Rickli H. Riesen W. Binggeli C. Duru F.. (2007). N-terminal pro-brain natriuretic peptide used for the prediction of coronary artery stenosis. Eur. J. Clin. Invest. 37, 18–25. 10.1111/j.1365-2362.2007.01731.x 17181563

[B57] Xu M. Liu P. P. Li H. (2019). Innate immune signaling and its role in metabolic and cardiovascular diseases. Physiol. Rev. 99, 893–948. 10.1152/physrev.00065.2017 30565509

[B58] Zelechowska P. Agier J. Kozlowska E. Brzezinska-Blaszczyk E. (2018). Mast cells participate in chronic low-grade inflammation within adipose tissue. Obes. Rev. 19, 686–697. 10.1111/obr.12670 29334696

